# Effect of Cryopreservation Method Supported with Biochemical Analyses in the Axillary Bud of Jewel Orchid, *Ludisia discolor*

**DOI:** 10.3390/plants11070879

**Published:** 2022-03-25

**Authors:** Hazirah Burkhan, Kirutika Selva Rajan, Suganthi Appalasamy, Ranjetta Poobathy, Bee Lynn Chew, Vanitha Mariappan, Sreeramanan Subramaniam

**Affiliations:** 1 School of Biological Sciences, Universiti Sains Malaysia (USM), Georgetown 11800, Penang, Malaysia; hazirah.b@student.usm.my (H.B.); kirutika28@gmail.com (K.S.R.); beelynnchew@usm.my (B.L.C.); 2 Department of Natural Resource and Sustainability, Faculty of Earth Science, Universiti Malaysia Kelantan (UMK), Locked Bag No. 100, Jeli 17600, Kelantan, Malaysia; suganthi.a@umk.edu.my; 3 School of Biological Sciences, Quest International University (QUIP), Ipoh 30250, Perak, Malaysia; ranjetta.poobathy@qiup.edu.my; 4 Centre of Toxicology and Health Risk Studies (CORE), Faculty of Health Sciences, Universiti Kebangsaan Malaysia (UKM), Kuala Lumpur 50300, Federal Territory of Kuala Lumpur, Malaysia; vanitha.ma@ukm.edu.my; 5 National Poison Centre, Universiti Sains Malaysia (USM), Georgetown 11800, Penang, Malaysia; 6 School of Chemical Engineering Technology, Universiti Malaysia Perlis (UNIMAP), Arau 02600, Perlis, Malaysia; 7 Centre for Chemical Biology, Universiti Sains Malaysia (USM), Bayan Lepas 11900, Penang, Malaysia

**Keywords:** axillary bud, cryopreservation, droplet-vitrification, *Ludisia discolor*, orchid, reactive oxygen species

## Abstract

This study investigated conserving an endangered terrestrial jewel orchid *Ludisia discolor,* using in vitro grown axillary buds. Excised segments of axillary buds (4–5 mm in length) were precultured on a modified Murashige and Skoog (MS) medium supplemented with 0.2 M sucrose for 24 h and osmoprotected in a loading solution for 20 min. Then, axillary buds were dehydrated in plant vitrification solution 2 (PVS2) for 10 min at 0 °C and incubated in liquid nitrogen for 1 h. Subsequently, axillary buds were rewarmed rapidly by dilution solution and transferred to a growth recovery medium supplemented with 0.05 µM melatonin, which led to an improved survival chance (16.67%) for cryopreserved *L. discolor*. The osmotic stress and the overproduction of reactive oxygen species (ROS) during cryopreservation stages may result in cryoinjuries and poor survival as increased levels of proline (5.51 µmol/g), catalase (85.64 U/g), peroxidase (565.37 U/g), and ascorbate peroxidase activities (12.19 U/g) were detected after dehydration, preculture, rewarming, and loading stage, respectively. Results obtained from this study indicate that further experimental designs which apply different PVS and exogenous antioxidants are needed for improved survival and regrowth of *L. discolor.*

## 1. Introduction

Orchidaceae is one of the most abundant and globally distributed flowering plant families [1], with some 972 species in 159 genera recorded in Peninsular Malaysia [2,3,4], of which at least 136 species were found in Penang Hill [5]. Ludisia belongs to a group of terrestrial orchids cultivated for their attractive ornamental leaves, commonly referred to as ‘Jewel Orchids’ [6]. These ground-dwellers found thriving on the damp forest floors are native to China and Southeast Asia, including Malaysia.

Many orchids are traded commercially for medicine, food, and ornamental plants [7,8,9]. Due to their symbiotic association with mycorrhizal fungi, specialised pollinators, and limited germination rates [10,11,12], most orchid species are narrowly distributed in specific habitats. They are steadily dwindling in their natural population through habitat loss and indiscriminate collection of wild orchids. A case study on orchid extinction in Malaysia was well documented by Go et al. [13].

Maintenance of in vitro plants collection by repeated subcultures is labour-intensive, costly and the risks of contamination and somaclonal variation in orchid materials increase with time [14,15]. Such drawbacks have led to safe and long-term conservation strategies through cryopreservation of organs and tissues that have made significant progress over the years [7,16,17]. Cryopreservation is the storage of biological materials at ultra-low temperature, typically liquid nitrogen (LN) at −196 °C [18]. All metabolic activities such as respiration and cellular division of biological materials (including seeds, stems apices, axillary and apical buds, and cell suspensions) are halted at such temperatures. Thus, the risks of somaclonal variation or genetic changes are reduced, which in principle enables indefinite conservation [19]. In practice, biological materials of commercially important crops threatened plants, and plants with horticultural importance survived the storage durations in LN for as short as 1–48 h [20,21] and as long as 1.5–28 years [20,22,23,24,25].

Vitrification-based techniques constitute one of the contemporary methods in preserving orchids’ biodiversity [7]. Vitrification refers to a phase transition from liquid water to solid glass instead of lethal ice crystals, forming within cells upon rapid cooling in LN temperatures [26]. During dehydration, as the amorphous glass occupies space in a cell, it may hamper cell deterioration, solute concentration, and pH alteration [19] while maintaining the high cell viscosity. Droplet-vitrification is one of the recent techniques being adopted and continuously refined in orchid cryopreservation [27] as it combines both rapid cooling and warming strategy with a mixture of high solute concentration of cryoprotectants (namely dimethyl sulfoxide [DMSO], ethylene glycol, and sugar such as glucose or sucrose) to inhibit ice nucleation and growth [28].

Vitrification-based techniques inflict various stressful conditions on the plant materials, resulting in impairment in growth after rewarming. The stresses that occurred included excision of plant materials, osmotic injury and dehydration following treatment with highly concentrated cryoprotectants, and freezing injury from sudden temperature changes. Consequently, oxidative damage occurs due to the increased accumulation of reactive oxygen species (ROS) [29,30]. ROS, including superoxide radical (O_2_^−^), hydrogen peroxide (H_2_O_2_), hydroxyl radical (OH^−^), and singlet oxygen (^1^O_2_), is produced in chloroplasts, mitochondria, and peroxisomes [31]. Under typical growth environments, there is an equilibrium between ROS production and protective antioxidants. However, overproduction of ROS in stressed cells will exhaust the capacity of the antioxidants in cellular repair processes, and biological deterioration can occur [32]. Hence, various precautions are taken to induce cell tolerance and regrowth ability, such as the inclusion of sugar in preculture media [33], the mixture of cryoprotectants, exposure duration, and temperature [34], the alteration in regrowth media with the addition of supplements [35,36], and light condition [37].

However, much uncertainty still exists about the harmful effects experienced by a cryopreserved cell or tissue during the osmo-cryoprotection freeze–thaw period and post-thaw recovery. An analytical tool such as biochemical analyses is efficient to provide insights into the effect of cryopreservation at tissue levels. Proline, catalase (CAT), peroxidase (POX), and ascorbate peroxidase (APX), which are part of the protective antioxidants that scavenge the excess ROS in plants, can be utilised as biomarkers indicating plant defence mechanisms in response to various oxidative stress encountered during cryopreservation stages [30,38].

This study sought to examine the effects of cryopreserving *L. discolor* orchid by droplet-vitrification technique. Subsequently, data obtained from the biochemical activities will ascertain the need for exogenous antioxidants during cryopreservation.

## 2. Results

### 2.1. Cryopreservation by Droplet-Vitrification Protocol

In this study, the feasibility of cryopreserving axillary buds of *L. discolor* based on the effect of pretreatment with sucrose (concentration and duration), dehydration (duration and temperature), and the presence of antioxidants in growth recovery was investigated based on the absorbance reading and percentage of survival.

**Figure 1:**
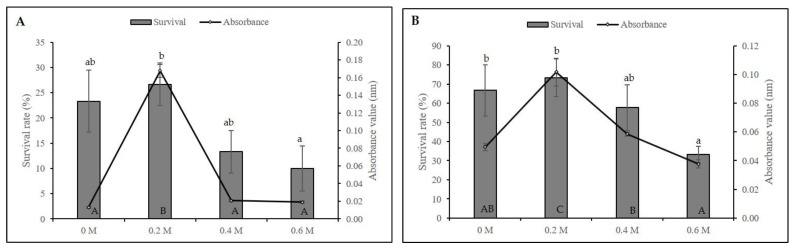
Effect of sucrose concentration during preculture at 24 h on the viability of (**A**) cryopreserved (+LN) and (**B**) non-cryopreserved (−LN) axillary buds before dehydration and LN immersion. Error bars represent standard errors. Means with different letters (upper case: Absorbance; lower case: Survival) are significantly different based on Duncan Multiple Range Test (DMRT) at *p* ≤ 0.05.

**Figure 2:**
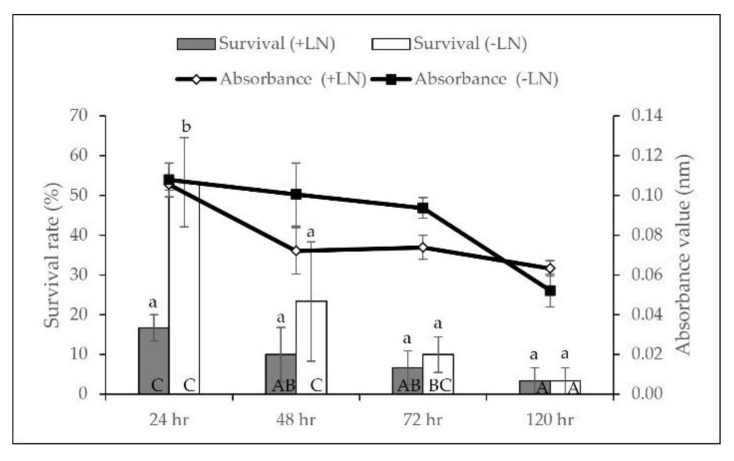
Effect of preculture duration in a 0.2 M sucrose on the viability of cryopreserved (+LN) and non-cryopreserved (−LN) axillary buds before dehydration and LN immersion. Error bars represent standard errors. Means with different letters (upper case: Absorbance; lower case: Survival) are significantly different based on Duncan Multiple Range Test (DMRT) at *p* ≤ 0.05.

The survival percentage for cryopreserved axillary buds ranged from 10–26.67% with axillary buds precultured in a 0.2 M sucrose showing the highest survivability with a significant difference in the absorbance value (A_490nm_: 0.168). By contrast, increasing sucrose concentration to 0.4 and 0.6 M resulted in 13% and 10% survival, respectively (Figure 1A). A similar trend was observed on non-cryopreserved axillary buds, decreasing the survivability of 33.33% (A_490nm_: 0.038) when precultured in 0.6 M sucrose (Figure 1B). We further optimised preculture duration with 0.2 M sucrose and found that axillary buds of *L. discolor* showed sensitivity towards sucrose. Prolonged exposure to sucrose negatively affected the survival of axillary buds with a sharp decrease to 3.33% (Figure 2). The non-cryopreserved axillary buds also showed the same pattern of viability.

**Figure 3:**
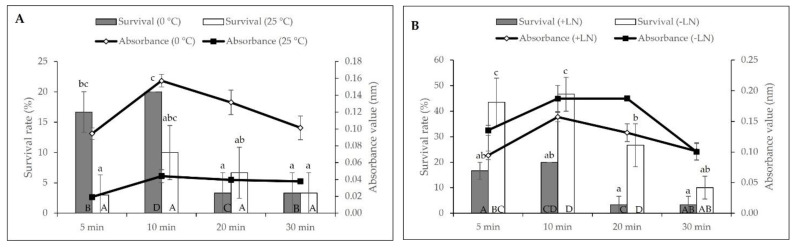
Effect of PVS2 dehydration duration on the viability of (**A**) cryopreserved (+LN) axillary buds at 0 °C and 25 °C and (**B**) cryopreserved (+LN) and non-cryopreserved (−LN) axillary buds at 0 °C. Error bars represent standard errors. Means with different letters (upper case: Absorbance; lower case: Survival) are significantly different based on Duncan Multiple Range Test (DMRT) at *p* ≤ 0.05.

Figure 3 showed the survival of axillary buds after dehydration with PVS2 for different durations at 0 and 25 °C. PVS2 incubated at 0 °C was associated with higher survival of axillary buds than PVS2 set at 25 °C. Cryopreserved axillary buds exposed to PVS2 for up to 10 min at 0 °C resulted in an increase of survival at 20% (A_490nm_: 0.157) with a significant reduction when incubated at 25 °C (Figure 3A). As exposure periods were prolonged to 30 min for both temperatures, the survival of axillary buds declined significantly. Figure 3B illustrated the varied response for both cryopreserved and non-cryopreserved axillary buds at 0 °C. Survival gradually increased from 43.33% (5 min) to 46.67% (10 min) before a decrease of 10% when the non-cryopreserved axillary buds were exposed to PVS2 at 0 °C. Therefore, dehydration with PVS2 at 0 °C for 10 min was chosen for the subsequent experiment.

**Figure 4:**
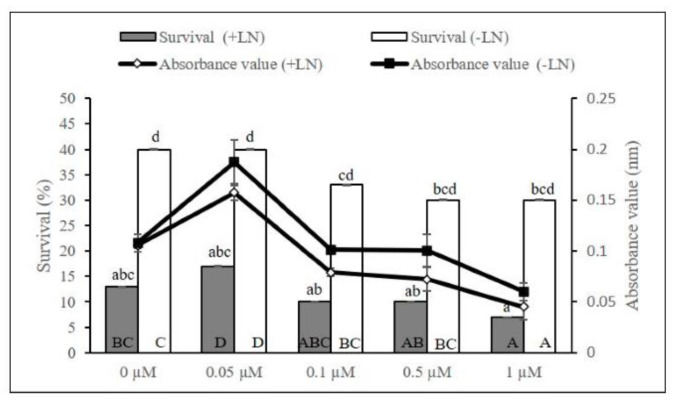
Effect of exogenous melatonin concentration on the viability of cryopreserved (+LN) and non-cryopreserved (−LN) axillary buds during the growth recovery stage. Error bars represent standard errors. Means with different letters (upper case: Absorbance; lower case: Survival) are significantly different based on Duncan Multiple Range Test (DMRT) at *p* ≤ 0.05.

The TTC absorbance values obtained from extracts of both cryopreserved (A_490nm_: 0.157) and non-cryopreserved (A_490nm_: 0.187) axillary buds were significantly different with the addition of 0.05 µM melatonin in growth recovery media (Figure 4). Although no significant difference in survival of cryopreserved axillary buds was observed, a growth recovery media fortified with 0.05 µM melatonin improved the chances of survival to 16.67% compared to 1.0 µM melatonin (6.67%). Comparably, better survival of non-cryopreserved axillary buds was recorded between 30–40%.

**Figure 5:**
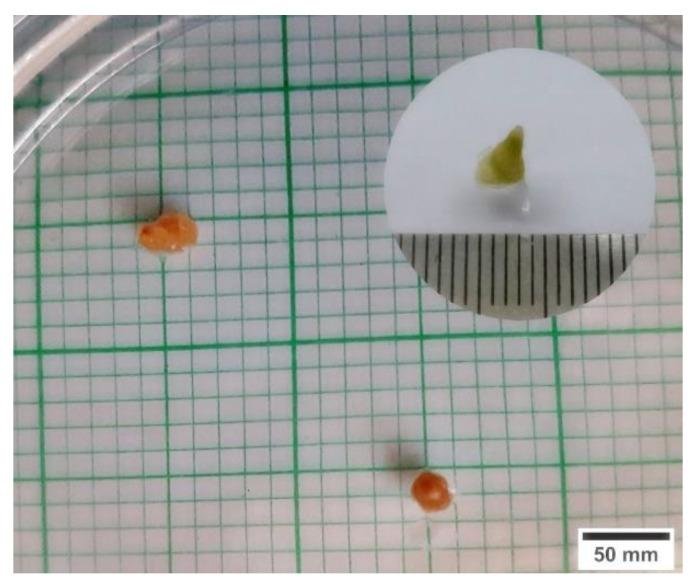
Visual observation of a viable axillary bud of *L. discolor* exhibiting a green colour (insert) compared with completely brown cryopreserved axillary buds obtained from a melatonin-treated culture after 3 weeks.

As depicted in Figure 5, the surviving cryopreserved axillary buds had viable green parts after culture on regrowth medium for 3 weeks, while browning was detected in dead cryopreserved.

### 2.2. Biochemical Analyses during Cryopreservation

**Figure 6:**
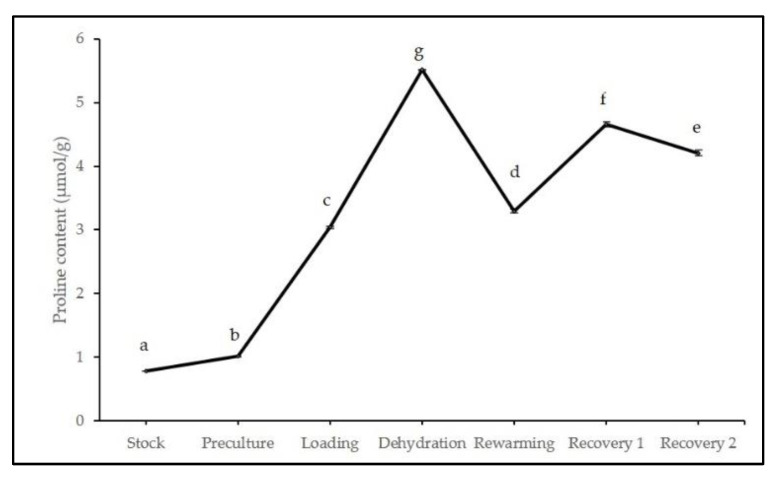
Proline content of cryopreserved axillary buds at different stages in cryopreservation. Error bars represent standard errors. Means with different letters are significantly different based on Duncan Multiple Range Test (DMRT) at *p* ≤ 0.05.

Different cryopreservation stages significantly affected the proline accumulation, total soluble protein, and enzyme activities (catalase, peroxidase, and ascorbate peroxidase) of treated axillary buds of *L. discolor*. Figure 6 revealed a gradual increase of proline content, which reached a peak during the dehydration stage (5.51 µmol/g) before a slight reduction of 40% after immersion in LN and rewarming. After three weeks in the growth recovery media (R2), the proline content was steadily reduced to 4.21 µmol/g.

**Figure 7:**
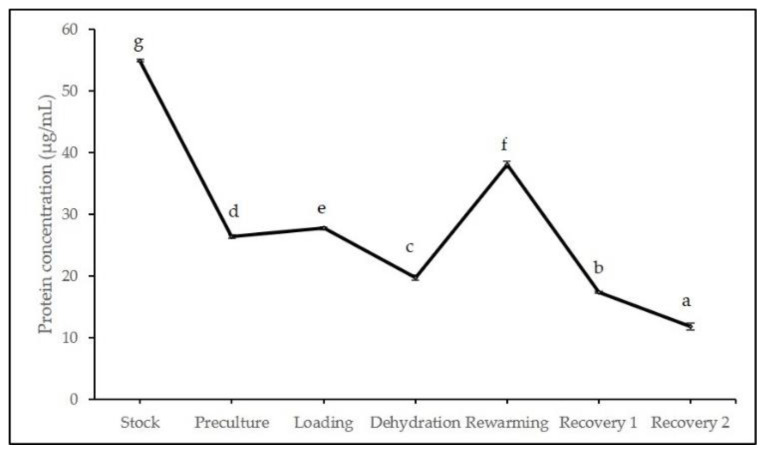
Total soluble protein content of cryopreserved axillary buds at different stages in cryopreservation. Error bars represent standard errors. Means with different letters are significantly different based on Duncan Multiple Range Test (DMRT) at *p* ≤ 0.05.

All stages in cryopreservation resulted in significant differences ranging from 11.88–54.95 µg/mL. Figure 7 indicated that stock culture contained the highest soluble protein content before continuing to drop following the early stage of cryopreservation (preculture, loading, and dehydration). After storage in LN for an hour and exposure to unloading solution, the soluble protein content sharply increased to 38.04 µg/mL before further declining in the recovery stage.

**Figure 8:**
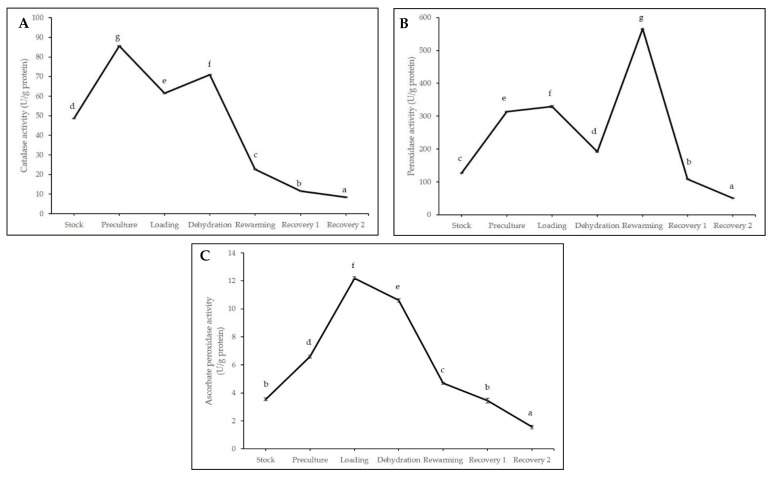
Antioxidant enzyme activities of (**A**) catalase; (**B**) peroxidase; and (**C**) ascorbate peroxidase of cryopreserved axillary buds at different stages in cryopreservation. Error bars represent standard errors. Means with different letters are significantly different based on Duncan Multiple Range Test (DMRT) at *p* ≤ 0.05.

Figure 8A–C showed the enzyme activities involved in protecting the cryopreserved axillary buds from injuries due to stress accumulated in each cryopreservation stage. The catalase activities peaked at the preculture (85.64 U/g) stage before a decrease during osmoprotection in the loading stage (Figure 8A). The catalase activities elevated in dehydration before gradually declined after incubation in LN with the lowest value of 8.43 U/g.

Figure 8B demonstrated fluctuated peroxidase activities. A significant increase between stock culture and preculture was observed before a slight decline during dehydration. Then, the peroxidase activities peaked at 565.37 U/g after LN exposure, followed by a steep drop during the recovery stage (R1).

The ascorbate peroxidase activities rose during the early stages of cryopreservation, with the highest value observed before the LN incubation (12.19 U/g) (Figure 8C). Afterwards, the enzyme activities decreased with no significant differences observed between the stock culture and when axillary buds were subjected for a day in a growth recovery media (R1). R2 recorded the lowest enzyme activities at 1.56 U/g.

## 3. Discussion

### 3.1. Cryopreservation by Droplet-Vitrification

Although cryopreservation methods including desiccation, preculture-desiccation, encapsulation-dehydration, vitrification, and droplet-vitrification have been developed for several genera of the Orchidaceae family [7,39,40,41,42], the cryopreservation method for Ludisia species has not been documented before. Therefore, this study reported the first attempt of long-term storage of *L. discolor*, using a droplet-vitrification method. Axillary buds were chosen as the plant materials due to the availability of the materials throughout the year and typically, plantlets regenerated via axillary buds or direct somatic embryogenesis are considered to be the most genetically uniform.

Notably, droplet-vitrification is one of the recent techniques adopted and continuously refined in orchid cryopreservation [27]. Success in this method requires optimising various parameters such as preculture (or not) on a sugar-enriched medium before incubating in the vitrification solutions that offer the advantage of rapid cooling and rewarming with a mixture of solute concentrations [43].

Sucrose preculture proves to be an essential step in enhancing the dehydration tolerance of *L. discolor* axillary buds to the subsequent cryo-treatment. The present study found the highest survival rate was achieved when axillary buds were precultured for 24 h in a 0.2 M sucrose before cryopreservation. Preliminary testing has been conducted for the preculture duration of less than 24 h (0 and 12 h), but negative results were demonstrated indicating the slight exposure to sucrose was not beneficial to the survivability of the jewel orchid. Taking the limited resources of stock materials into consideration, the duration of 24 h was chosen for the subsequent experiments.

By contrast, a progressive increase of sucrose concentration (beyond 0.2 M) and prolonged exposure resulted in a low survival rate. This suggests a possibility of sucrose over-accumulation in the cytoplasm, causing an imbalance of carbohydrate in the cellular state of cryopreserved explant and causing excessive dehydration and toxicity [44]. This is consistent with *Brassidium* Shooting Star orchid, whereby elevated sucrose concentration beyond 0.25 M negatively affected the viability of cryopreserved PLBs [45]. In cryopreserving protocorm-like bodies (PLBs) of *Cymbidium* Twilight Moon ‘Day Light’, sucrose at low concentration was found to be exceptional compared to the other osmoticum type, including different concentrations of mannose, polyethylene glycol (PEG-6000), and DMSO [46]. For *Vanilla planifolia*, apices treated with 0.3 M sucrose for one day resulted in 30% survival [47]. In contrast, PLBs of *Aranda* Broga Blue required 0.2 M sucrose for three days [48]. However, Popova et al. [7] concluded that most orchids favoured preculturing at 0.4–0.75 M for 1–2 days, including orchid hybrids of *Brassidium* Fly Away [39] and *Dendrobium* Bobby Messina [49]. For *Cymbidium eburneum* and *C. hookeranium*, exposure to 0.7 M sucrose was only required for 20 h [50]. Similarly, increased viability was recorded for orchid hybrid *Ascocenda* for sucrose treatment at 0.5 M for 18 h. Overall, existing literature agreed with the general notion that genotypic variations contribute to the varying survival rate due to sample heterogeneity. Thus, optimising the preculture condition is critical for a successful orchid cryopreservation protocol.

This study showed the duration and temperature of PVS2 treatment significantly influenced the survival of cryopreserved axillary buds of *L. discolor*. PVS2 contains a mixture of vitrification solutions including glycerol, ethylene glycol, and DMSO that supports the transition of liquid water to the glassy state during the rapid freezing and thawing steps of cryopreservation, thus avoiding the lethal formation of ice crystals [51]. With adequate exposure, these concentrated solutes can dehydrate, penetrate, and induce adaptation towards freezing within the cryopreserved explant [34]. The temperature and length of application can influence the effectiveness, toxicity, and permeability of vitrification solutions. Consequently, these considerations may need to be evaluated case-by-case basis [52,53].

Present work substantiates previous findings in the literature whereby overexposure to vitrification solutions in an elevated temperature is associated with lower explants survival [44,54,55]. With *Dactylorhiza fuchsii* protocorms, 1 h of PVS2 treatment at 0 °C was necessary to stimulate growth [56]. Incubation time and temperature for PVS2 usually differ with plant species, as recorded in previous literature. Within Cymbidium species, varied responses were documented when vitrification technique was employed on different explants such as PLBs of *C. finlaysonianum* and *Cymbidium* Twilight Moon ‘Day Light’ endured well in 60 min and 80 min of PVS2, respectively, on ice. In contrast, optimum durations were documented for seeds to be 30 min for *C. finlaysonianum* and 60 min for *C. goeringii* and *C. macrorhizon* [42,46,57]. By contrast, increased temperature is suitable for cryopreserved orchids to be sufficiently dehydrated in *Dendrobium cruentum*, *D. signatum*, and *Paphiopedilum niveum* [58,59,60,61].

Abiotic stressors during cryopreservation (explant excision, osmotic injury, tissue dehydration, and temperature changes) promote an overproduction of reactive oxygen species (ROS) that causes oxidative damage [29,62]. Moderate ROS production is necessary for secondary signalling molecules [63]. However, under stressed conditions, Møller et al. [64] elaborated that ROS such as _1_O^2^, O_2_^−^, H_2_O_2_, and OH^−^ can severely damage the membrane structures and other cell components by incorrect timing of programmed cell death. Scavenging these surplus radicals may achieve tolerance to such harmful effects [65]. Published reports have employed antioxidants such as ascorbic acid, glutathione, and melatonin to directly remove the ROS and protect cellular functions, thus improving the chances of survival [35,36,62,66,67,68]. Studies describing the protective role of melatonin and enhancement in the recovery of cryopreserved shoot tips of American elm and yam were documented [66,69]. Similarly, melatonin has been found to act as a direct and indirect antioxidant with both lipophilic and hydrophilic properties, and therefore, it may serve as a highly potent antioxidant and protects lipids against peroxidation [44,70]. The small size of melatonin’s molecule allows it to migrate easily between the compartments of cells protecting them from excessive ROS levels. Melatonin showed much higher antioxidant activity than vitamins C, E and K. This is maybe due to better penetration of melatonin into the cell compartments while vitamins are capable only of selective migration [71]. Contrary to expectations, present findings showed that the addition of melatonin during the recovery stage did not promote the post-thaw recovery of cryopreserved axillary buds. The reason for this rather contradictory result is still not entirely clear, but a physiological decline could be attributed to ROS production that surpasses the capacity of melatonin in the cell, thereby reducing the ability of the antioxidant to function, resulting in only 16.67% of survivability for cryopreserved axillary buds and 40% for non-cryopreserved control axillary buds.

Overall, there is a lot of variation in cryopreservation success among different species. Additionally, there is also an important methodological difficulty, as experimental protocol steps tested on one or a few genotypes in one genus may not work with others [7]. Given the wide diversity of orchids and the progress in orchid cryopreservation, Das et al. [72] concluded that it would be useful to investigate any correlations between taxon and explants and cryopreservation procedures. Due to the small number of orchid species that have been cryopreserved, this type of comparison is challenging. Given the current state of research, it is more beneficial to consider the different types of cryopreservation procedures that are available and their efficacy on the various types of explants that can be stored.

### 3.2. Biochemical Analyses during Cryopreservation

Biochemical analyses were performed as an indication of oxidative stress encountered during cryopreservation. There is a general consensus that in response to different stresses, plants accumulate large amounts of different types of compatible solutes. Compatible solutes are highly soluble, low molecular weight organic compounds that are not normally toxic at high cellular concentrations. These solutes protect plants from stress by contributing to cellular osmotic adjustment, ROS detoxification, protection of membrane integrity, and enzyme/protein stabilization [73,74]. These include proline, sucrose, polyols, trehalose, and quaternary ammonium compounds (QACs) such as glycine betaine, alanine betaine, proline betaine, and pipecolate betaine [75].

In the present work, proline contents significantly increased during the early stages of cryopreservation and peaked during dehydration before being reduced after LN exposure. This signified that stressful dehydration conditions resulted in an overproduction of proline in cryopreserved axillary buds. Similarly, cryopreserved *Dendrobium* Sabin Blue exhibited the highest proline level after dehydration with PVS2 treatment [30]. According to the literature [76,77], these increments in the proline contents during stress function as an osmoprotectant and antioxidant. Several studies have explored the occurrence of amino acid such as proline typically in the cytoplasm and contribute to stress tolerance by behaving as a molecular chaperon that maintains the membrane integrity, alters cell osmotic pressure, and remove ROS [74,78,79].

Fluctuated outcomes were observed in the total soluble protein and all antioxidant enzyme activities (CAT, POX, and APX) involved in protecting cryopreserved axillary buds of *L. discolor* from injuries to stress accumulated in each cryopreservation stage. Nevertheless, this does not necessarily affect the cell survival following cryopreservation as the proteins involved in combating stress could be synthesized in a smaller amount compared to the amount during the plants grown under normal conditions [38]. Drastic reduction of total soluble protein was observed during the recovery stage after storage in LN, indicating protein degradation due to high ROS production. On the other hand, cryopreserved axillary buds enhanced stress tolerance by increasing the synthesis of enzymatic antioxidants: CAT and APX (initial stages of cryopreservation) and POX (immediately after LN exposure followed with rewarming) that breaks down and remove the excess free radicals. The increased expression of antioxidants among the critical stages of cryopreservation may be due to the high production of H_2_O_2_, which in return triggers antioxidant activities as ROS scavengers [30,80]. However, the reduced expressions of antioxidant enzyme activities reflected the cryopreserved explants’ inability to combat the accumulation of ROS, which in return led to the poor survival of explants and was associated with tissue browning as reported by Rahmah et al. [81]. Trchounian et al. [82] added that the capacity of exogenous antioxidants to detoxify ROS and maintain homeostasis varies on the growth stage of the explants, genotypic differences to osmotic stress, and stress intensity levels [82].

## 4. Materials and Methods

### 4.1. Plant Material

**Figure 9:**
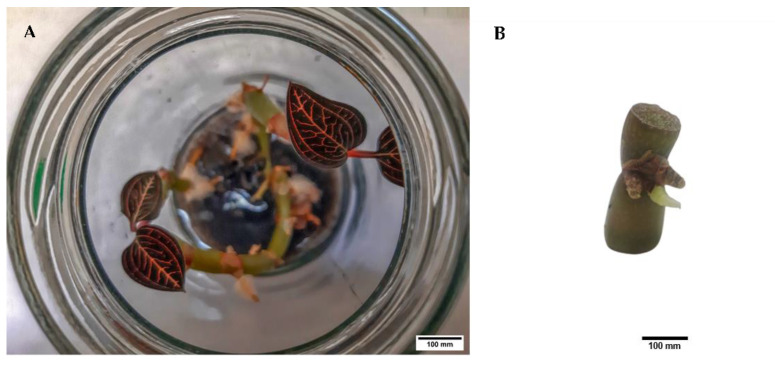
(**A**) In vitro stock culture of *L. discolor* propagated from nodal segments and (**B**) excised node with axillary bud present.

In vitro stock culture of *L. discolor* (Figure 9) was obtained from Laboratory 101, Industrial Biotechnology Research Laboratory, School of Biological Sciences, Universiti Sains Malaysia.

Nodal segments of *L. discolor* were aseptically cultured to initiate axillary buds for cryopreservation study. Half-strength semi-solid Murashige and Skoog (1962) [83] medium (MS) fortified with 0.2% (*w*/*v*) activated charcoal, 8% (*w*/*v*) Mas banana cultivar homogenate, 3.5 g/L Gelrite^TM^, 1.0 mg/L 1-naphthaleneacetic acid (NAA), and 0.1 mg/L thidiazuron (TDZ) was used to propagate *L. discolor* [6]. The cultures were maintained at a temperature of 24 ± 2 °C with a relative humidity of 51 ± 2% under cool white light-emitting diode (LED) (17 µmol/s) with 16/8 h of light/dark regime (standard growth condition). Micropropagation of the in vitro stock cultures was performed at a 12-week interval.

### 4.2. Media Preparation

All media used in the study were supplemented with a half-strength MS medium. The preculture media were enriched with sucrose concentrations (0.0, 0.2, 0.4, and 0.6 M), while the loading solution contained 0.4 M sucrose and 2.0 M glycerol (J.T. Baker, Phillipsburg, NJ, USA). The PVS2 consisted of 30% (*w*/*v*) glycerol, 15% (*w*/*v*) ethylene glycol (Friendemann Schmidt, Washington, USA), 15% (*w*/*v*) DMSO (Qrec, Rawang, Selangor, Malaysia), and 0.4 M sucrose [84], and the unloading solution contained 1.2 M sucrose. The growth recovery media were half-strength semi-solid MS enriched with 3% sucrose and 2.75 g/L Gelrite^TM^. The pH was all adjusted to 5.7–5.8 by adding 1 N sodium hydroxide (NaOH) or 1 N hydrochloric acid (HCl) before autoclaving at 121 °C for 15 min (Tomy High-Pressure Steam Sterilizer ES-315, Tokyo, Japan). Melatonin (0, 0.05, 0.10, 0.50, and 1.0 µM) (Sigma-Aldrich, Saint Louis, MO, USA) was prepared and pH was adjusted to 5.7 before being used. Melatonin was added to the sterilized growth recovery media using membrane filters (0.45 μm, Nalgene, Bellevue, WA, USA). All chemicals are manufactured from Duchefa Biochemie (Haarlem, The Netherlands) unless specified otherwise.

### 4.3. Cryopreservation Method

Axillary buds of *L. discolor* (4–5 mm) were used as a starting material for cryopreservation using the droplet-vitrification method adapted from Khor et al. [48].

The excised axillary buds were precultured in a half-strength semi-solid MS medium supplemented with sucrose (0, 0.2, 0.4, and 0.6 M) at various durations (24, 48, 72, and 120 h). Next, the axillary buds were osmoprotected with 1.5 mL of loading solution for 20 min at 25 °C. Then, the axillary buds were dehydrated in PVS2 for 20 min at 0 °C. Five minutes before the end of the dehydration treatment, the axillary buds were placed on an aluminium foil strip (1 cm × 3 cm) in a droplet of PVS2 before direct immersion in LN (MVE lab 20, MVE Bio-Medical Division, Chart Industries, Inc., Ball Ground, GA, USA). The aluminium foils (secured in a cryotube) were then directly plunged in LN and maintained for at least 1 h. Next, the aluminium strips were removed from the cryotube and quickly rewarmed in 10 mL of unloading solution at 25 °C. Swirled the solution for a few seconds until the axillary buds detached from the strips and held for another 20 min at 25 °C.

The best results from the preculture durations and temperatures were selected for subsequent experiments: PVS2 for 5, 10, 20, and 30 min (at 0 and 25 °C). A similar method was performed to melatonin concentrations (0, 0.05, 0.10, 0.50, and 1.0 µM) during growth recovery.

Following the cryopreservation protocol, the axillary buds were transferred onto sterile filter paper affixed on half-strength MS regrowth media and incubated in the dark overnight. For the first week, the axillary buds were kept in complete darkness followed by one week under dim light conditions (3 μmol/s) and subsequently incubated under standard growth conditions of 16-h photoperiod.

Control axillary buds were treated with all the solutions, but not immersed in LN (non-cryopreserved; −LN).

#### Determination of the Axillary Bud’s Survival Rate

The survival of both control and cryopreserved axillary buds was assessed three weeks after the cryopreservation protocol using 2,3,5-triphenyltetrazolium chloride (TTC) spectrophotometric analysis at 490 nm (U-1900 UV-VIS Spectrophotometer 200V, 3J0-0003, Hitachi, Tokyo, Japan). The TTC assay method was adapted from prescribed protocols [85,86] with slight modifications. The axillary buds from the regrowth media were transferred into universal bottles containing 2 mL of TTC solution: 0.6% (*w*/*v*) TTC (Sigma-Aldrich, Missouri USA) and 0.05% (*v*/*v*) Tween 80 (R&M Chemicals, London, UK) in a buffer solution consisting of 0.05 M of both disodium hydrogen phosphate dihydrate (Na_2_HPO_4_·2H_2_O) and potassium dihydrogen phosphate (KH_2_PO_4_) (R&M Chemicals, London, UK) at pH 7.4 [87]. The axillary buds were incubated in the dark for 24 h.

After incubation, axillary buds were subjected to visual inspection of the surface stained with formazan using a stereoscopic dissecting microscope (Olympus, Tokyo, Japan), with any red-stained evidence axillary buds confirming the existence of viable cells as evaluated by Poobathy et al. [38].

For the spectrophotometric-TTC assay, the residual TTC solution in the universal bottle was removed, and the axillary buds were rinsed three times with 3.5 mL of distilled water. Then, the formazan colour in the axillary buds was extracted with 7 mL of 95% ethanol (HmbG Chemicals, Hamburg, Germany) in a water bath at 80 °C for one hour. The extract was cooled, and a spectrophotometer analysed the reading absorbance of formazan colour at 490 nm wavelength against a blank of 95% ethanol.

The survival percentage was confirmed by observation after 3 weeks of culture on the regrowth medium. All and partially green axillary buds were evaluated for survival while completely brown or white denoted as dead.

### 4.4. Biochemical Analyses

Axillary buds were collected for enzyme extraction at various cryopreservation stages adapted from Mubbarakh et al. [67] as follows:

1. Axillary buds excised from in vitro plants (control);
2. Preculture on medium containing 0.2 M sucrose;
3. Osmoprotection by loading solution;
4. Dehydration with PVS2 for 10 min;
5. Rapid cooling and rewarming;
6. Recovery after 1 day on growth medium with 0.05 µM melatonin (Recovery 1);
7. Recovery after 3 weeks on growth medium with 0.05 µM melatonin (Recovery 2).

#### 4.4.1. Proline Analysis

The proline content was determined according to Bates et al. [88]. About 100 mg of axillary buds were macerated using mortar and pestle and placed in a 25 mL test tube. Following that, 15 mL of 80% (*v*/*v*) ethanol was added into the test tube and was incubated at 60 °C water bath for 30 min. The extract was filtered, and proline content (µmol/g) was measured with acid ninhydrin solution at A_520nm_ to obtain the absorbance reading, and data were compared with the proline standard curve.

#### 4.4.2. Total Soluble Protein Content

The Bradford method [89] was employed to determine the protein content of the collected axillary buds. Bradford reagent was prepared by diluting Coomasie Brilliant Blue G-250 (Amresco, Solon, Ohio, USA), 95% (*v*/*v*) ethanol, and 85% phosphoric acid (J.T. Baker, Phillipsburg, New Jersey, USA) with distilled water. The protein reagent was filtered to eliminate impurities until the solution turned brown. The solution was maintained in an amber bottle and refrigerated at 4 °C.

Axillary buds weighing 100 mg were ground in an iced cold mortar and homogenised with 0.6 mL of enzyme extraction buffer solution. Enzyme extraction buffer solution was made of 100 mM potassium phosphate buffer (pH 7.8), 2 mM ethylenediaminetetraacetic acid and 2 % (*w*/*v*) polyvinylpyrrolidone [90]. The mixture was centrifuged at 15 000× *g* for 20 min (Gyrozen, Model 1524, Gimpo, South Korea). The resulting supernatant was stored overnight under a −40 °C freezer.

The extract solution with a volume of 0.1 mL was transferred into 1 mL of Bradford reagent and vortexed. Bradford reagent was filtered with a 0.45-μm syringe filter before adding extract solution. Soluble protein concentrations (µg/mL) were taken at A_595nm_ and compared with the standard bovine serum albumin (BSA).

#### 4.4.3. Catalase (CAT) Assay

The CAT enzyme activity was analysed according to Sánchez-Rojo et al. [91]. The protein extract was obtained by homogenising the chopped axillary buds (100 mg) and an enzyme extraction buffer solution. The reaction mixture contained 30 µL protein extract, 50 mM potassium phosphate buffer (pH 7.0), and an addition of 30 mM H_2_O_2_ (R&M Chemicals, London, UK) to initiate the reaction. The decomposition of H_2_O_2_ was analysed, and the absorbance was recorded at A_240nm_ for 3 min. The calculation for the enzyme activity in U/g was performed according to the formula derived from Flocco and Giulietti [92], as demonstrated by Poobathy et al. [38].

#### 4.4.4. Peroxidase (POX) Assay

The POX assay was evaluated following the protocol based on Sánchez-Rojo et al. [91] and Antony et al. [30]. The protein extract was obtained by grinding 100 mg of axillary buds in an iced cold mortar and homogenised with an enzyme extraction buffer solution. The total reaction mixture of 3 mL contained 50 mM sodium phosphate (pH 7.0), 7.2 mM guaiacol, 6.86 mM H_2_O_2_ and was initiated by 30 µL of protein extract. The reaction progress was measured by the increment in absorbance at A_470nm_ for 3 min. The enzyme activity (U/g) was calculated according to Flocco and Giulietti [92].

#### 4.4.5. Ascorbate Peroxidase (APX) Assay

The APX enzyme activity was adapted from Elavarthi and Martin [93] and Rahmah et al. [81]. Axillary buds weighing 100 mg were ground using a pre-cooled mortar and homogenised with an enzyme extraction buffer solution. The 3 mL assay mixture contained 50 mM potassium phosphate (pH 7.0), 0.5 mM ascorbic acid (Sigma-Aldrich, USA), and 30 µL of protein extract. The reaction was initiated with 0.5 mM H_2_O_2_ and subsequently monitored at A_290nm_ for 3 min. The ascorbate peroxidase activity (U/g) calculation was based on Flocco and Giulietti [92].

### 4.5. Statistical Analysis

The cryopreservation treatments consist of six replicates exposed to LN (+LN) and six without LN (−LN), each containing five axillary buds. Experiments were performed according to a randomised complete block design. Each biochemical analysis comprises six replicates consisting of enzyme extraction of 100 ± 5 mg of axillary buds per treatment. Means were analysed by one-way analysis of variance (ANOVA) followed with Duncan Multiple Range Test (DMRT) with the probability value set at 0.05 using IBM SPSS Statistics for Windows, version 25 (IBM Corp., Armonk, NY, USA). The results were expressed as means ± SE (standard error).

## 5. Conclusions

Challenges remain regarding the slow growth of this jewel orchid. While less than 30% of survival level was obtained, the possibility of cryopreserving *L. discolor* with careful planning and testing could be ensured. Biochemical profiling as a stress indicator shows adaptive responses of the antioxidant system, which may be correlated with elevated ROS production that resulted in low viability of tissues after cryopreservation procedures. However, this research is in the initial stage, and further investigations, including optimising different vitrification solutions and incubation periods and applying antioxidants at various stages of cryopreservation, are warranted to improve this protocol and stimulate the regrowth of cryopreserved axillary buds. Histological approaches can also be considered to monitor the extent of cellular injuries inflicted during each cryopreservation stage.

## Data Availability

Data are contained within the article.
